# Supplementary material for: ChatGPT as a Tool for Biostatisticians: A Tutorial on Applications, Opportunities, and Limitations
Source: Stat Med. 2025 Oct 23;44(23-24):e70263. doi: 10.1002/sim.70263 (PMC12548020; doi:10.1002/sim.70263)
Supplement: Supplementary file 1 — Data S1. Supporting Information. [file SIM-44-0-s001.zip › Supplementary Materials Additional.zip!/3.1_Systematic_Reviews_and_Meta_aspects/3.1_Data Extraction - o4-mini - Chat 5.pdf]

OpenAI

Use Case Meta-Analysis - Data Extraction - o4-mini - Chat 5

Rudloff et al. - 2009 - Concurrent lobular neoplasia increases the risk of ipsilateral breast cancer recurrence in situ treated with breast-conserving therapy.pdf
PDF

Vidali et al. - 2012 - Conservative treatment of breast ductal carcinoma in situ results of an Italian multi-institutional.pdf
PDF

The aim is to perform a meta-analysis on total ipsilateral local recurrence rates at 10 years in cases of ductal carcinoma in situ.

Data inclusion rules are: To ensure the validity of the follow-up data, the results should be as close to 10 years follow-up (minimum) as the information in each study allows. If there is more than one publication from an institution or group, the most recent study with appropriate follow-up will be used to extract 10-year data.

Please provide from each of the two attached studies:

- the rate of ipsilateral local recurrence for conservative surgery with additional radiotherapy (no other interventions or drugs allowed) after 10 years

- the number of eligible patients

- period of data collection

- duration of follow-up

- type of study

- age of patients (mean or median, maximum and minimum)

- treatment modalities

For this task you have access to up-to-the-minute web retrieval to find adequate approaches for data extraction. When answering the task above please proceed as follows:

1. Fetch the most relevant sources.

2. Show your step-by-step reasoning.

3. At the end, give me a concise answer citing the sources you used.

4. Please provide a dataframe with one entry for each study, and column names "study", "rate", "n_patients", "period", "follow_up", "study_type", "age", and "treatment".

Step-by-Step Reasoning

1. **Identify the two studies and retrieve key metadata.**
   - **Rudloff et al. (2009)** is a single-institution retrospective cohort of 294 DCIS patients treated with breast-conserving therapy (BCT) between 1991–1995 .
   - **Vidali et al. (2012)** is a multi-institutional retrospective study of 586 DCIS patients treated between February 1985–March 2000 .
2. **Extract the 10-year ipsilateral local recurrence rate for patients receiving conservative surgery plus radiotherapy.**
   - **Rudloff et al.** report a 10-year local recurrence rate of 11.9% for women who underwent surgery and received radiation therapy .
   - **Vidali et al.** report a 10-year actuarial local recurrence risk of 9.6% .
3. **Determine the number of eligible patients.**
   - **Rudloff et al.**: 91 of 294 patients received adjuvant radiation after breast-conserving surgery .
   - **Vidali et al.**: 586 patients met inclusion criteria and underwent conservative surgery + RT .
4. **Record the period of data collection.**
   - **Rudloff et al.**: 1991–1995 .
   - **Vidali et al.**: February 1985–March 2000 .
5. **Extract duration of follow-up.**
   - **Rudloff et al.**: median follow-up was 11 years (range 0–16 years) .
   - **Vidali et al.**: median follow-up was 136 months (≈11.3 years; range 16–292 months) .
6. **Classify study type.**
   - **Rudloff et al.**: retrospective cohort analysis .
   - **Vidali et al.**: retrospective multi-institutional study .
7. **Collect patient age data.**
   - **Rudloff et al.**: median age 55 years (range 26–89) .
   - **Vidali et al.**: median age 55 years (range 29–84) .
8. **Summarize treatment modalities.**
   - **Rudloff et al.**: breast-conserving surgery plus external-beam whole-breast radiotherapy; no cytotoxic chemo; 6.1% received tamoxifen (excluded per task criteria) .
   - **Vidali et al.**: conservative surgery (quadrantectomy/tumorectomy/wide excision) followed by whole-breast RT (median dose 50 Gy; boost in 50% at median 10 Gy) .

Concise Answer

study	rate (10-yr ipsilateral recurrence)	n_patients	period	follow_up	study_type	age (median, range)	tre
Rudloff et al. (2009)	11.9%	91	1991–1995	median 11 yr (0–16 yr)	retrospective cohort analysis	55 yr (26–89 yr)	BCT: cons surg exte RT o
Vidali et al. (2012)	9.6%	586	Feb 1985–Mar 2000	median 136 mo (16–292 mo) ≈11.3 yr	retrospective multi-institutional study	55 yr (29–84 yr)	cons surg who RT (boos

Quellen
